# Hidden Costs of Hospital Based Delivery from Two Tertiary Hospitals in Western Nepal

**DOI:** 10.1371/journal.pone.0157746

**Published:** 2016-06-16

**Authors:** Jeevan Acharya, Nils Kaehler, Sujan Babu Marahatta, Shiva Raj Mishra, Sudarshan Subedi, Bipin Adhikari

**Affiliations:** 1 La Grandee International College, Pokhara University, Pokhara, Nepal; 2 Sandefjord Helsepark, Skiringssalveien 20, Sandefjord, Norway; 3 Department of Community Medicine, Manmohan Memorial Medical College and Teaching Hospital, Kathmandu, Nepal; 4 School of Population Health, University of Western Australia, Crawley, Western Australia, Australia; 5 School of Health and Allied Sciences, Pokhara University, Kaski, Nepal; 6 Mahidol-Oxford Tropical Medicine Research Unit, Faculty of Tropical Medicine, Mahidol University, Bangkok, Thailand; Public Health Agency of Barcelona, SPAIN

## Abstract

**Introduction:**

Hospital based delivery has been an expensive experience for poor households because of hidden costs which are usually unaccounted in hospital costs. The main aim of this study was to estimate the hidden costs of hospital based delivery and determine the factors associated with the hidden costs.

**Methods:**

A hospital based cross-sectional study was conducted among 384 post-partum mothers with their husbands/house heads during the discharge time in Manipal Teaching Hospital and Western Regional Hospital, Pokhara, Nepal. A face to face interview with each respondent was conducted using a structured questionnaire. Hidden costs were calculated based on the price rate of the market during the time of the study.

**Results:**

The total hidden costs for normal delivery and C-section delivery were 243.4 USD (US Dollar) and 321.6 USD respectively. Of the total maternity care expenditures; higher mean expenditures were found for food & drinking (53.07%), clothes (9.8%) and transport (7.3%). For postpartum women with their husband or house head, the total mean opportunity cost of “days of work loss” were 84.1 USD and 81.9 USD for normal delivery and C-section respectively. Factors such as literate mother (p = 0.007), employed house head (p = 0.011), monthly family income more than 25,000 NRs (Nepalese Rupees) (p = 0.014), private hospital as a place of delivery (p = 0.0001), C-section as a mode of delivery (p = 0.0001), longer duration (>5days) of stay in hospital (p = 0.0001), longer distance (>15km) from house to hospital (p = 0.0001) and longer travel time (>240 minutes) from house to hospital (p = 0.007) showed a significant association with the higher hidden costs (>25000 NRs).

**Conclusion:**

Experiences of hidden costs on hospital based delivery and opportunity costs of days of work loss were found high. Several socio-demographic factors, delivery related factors (place and mode of delivery, length of stay, distance from hospital and travel time) were associated with hidden costs. Hidden costs can be a critical factor for many poor and remote households who attend the hospital for delivery. Current remuneration (10–15 USD for normal delivery, 30 USD for complicated delivery and 70 USD for caesarean section delivery) for maternity incentive needs to account the hidden costs by increasing it to 250 USD for normal delivery and 350 USD for C-section. Decentralization of the obstetric care to remote and under-privileged population might reduce the economic burden of pregnant women and can facilitate their attendance at the health care centers.

## Introduction

In Nepal, a poor household woman faces a significant economic impediment in accessing health services during delivery due to hidden costs. A normal delivery for a woman from low to middle income family costs about 3 months of a household income compared to just over a month in a high income family [1]. Maternal mortality rate (MMR) and poverty have long been found to be closely associated [2].

The Maternal Mortality Rate in Nepal was estimated to be 258 deaths per 100,000 live births [3]. Under-utilization of health care services is one of the main contributing factors to high MMR in Nepal. Only 28% of babies are delivered at health centers while the MDG (Millennium Development Goal) goal of 60% is a long way to reach. Furthermore, a single ANC (Antenatal Care) visit during pregnancy shows high discrepancy between urban (88% attended at least one ANC care) and rural households (55% attended at least one ANC care) in utilizing health services in Nepal [4].

Nepal has formulated the national policies and plans to enhance the delivery of basic and essential health services to the general public. In 2008/9, the Government of Nepal (GoN) introduced the provision of free health service program to the general public through all governmental health care institutions and health workers. Under free health service scheme, GoN provides up to 40 essential medicines for free, through district level health facilities [5].

The drug availability in health facilities, especially the contraceptives, maternal and child health commodities, and selected essential drugs have improved the overall health care. However, there are several challenges for its implementation, of those are the dependence of the budget to donors (42% of the free health care is funded by donors), insufficient services to ensure the universal access with desired quality, limited human resources and lack of co-ordination between central and peripheral health facilities [5].

The government of Nepal started a Maternal Incentive Scheme in 25 least developed districts to encourage women to deliver babies at hospitals in 2005 which was later revised as “Safe motherhood program” in 2007 [6]. This program constituted the costs of transportation to health center for mothers, free institutional delivery and the incentive to a health worker [6, 8]. The Safe motherhood program provides the financial incentive (transportation incentive) depending upon the different geographical regions (5 USD for Terai, 10 USD for Hills and 15 USD for Mountain). In addition to free delivery at public health facilities, remuneration (10 USD for normal delivery at health facility with <25 beds, 15 USD for normal delivery at health facility with >25 beds, 30 USD for complicated delivery and 70 USD for caesarean section delivery) of hospital costs are provided depending upon the type of delivery and health facility. Additionally, health workers are provided incentive for each delivery (2 USD for delivery at home and 3 USD for delivery at institution) conducted [7]. A nationwide survey found that the safe motherhood program was unable to increase the health care uptake by pregnant women especially in rural regions [8]. The reasons for the lack of uptake of health facilities in rural regions were mostly attributed to geographical barriers, however, financial burdens especially the hidden costs were not explored in this study [8].

In Nepal, bulk of core costs (registrations, consultations, drugs and bed costs) are officially free of charge but the overall maternity care has been found to be expensive for families because of hidden costs which includes the purchase of drugs from private pharmacies, food costs, transportation costs, communication expenses (accessory communication need while being away from home), loss of days at work by both the patient and the patient’s accompaniers, unaccounted ancillary treatments, wasted drugs and laboratory tests [9–14]. Hidden costs are not physically recorded for any good or service provided but are an implicit in the overall expenditure [15]. Hidden costs were found to be the main reason for reluctance in Nepalese women to attend hospitals for deliveries despite free maternity services [16].

Among many other reasons, economic constraint faced by rural women attending the urban health centers possesses a significant impact on utilization of health services [16]. It is not enough to view hospital based delivery from provider’s perspective alone. An account of hidden expenditure for pregnant women to attend urban hospitals during delivery can reflect the real scenario of economic burden for pregnant women [17]. Rare studies in the past have been conducted in Nepal to explore the extent of hidden costs for pregnant women attending hospitals and the factors associated with it. Studies about hidden costs especially during pregnancy in a context of least developed countries such as Nepal can help health policy makers to direct the necessary programs to improve hospital attendance that might ultimately improve mother and child health (MCH). The main aim of this study was to estimate the hidden costs during hospital delivery and to determine the factors associated with hidden cost of hospital based delivery.

## Methods

### Study design

This study was a cross-sectional study estimating the hidden costs of hospital based delivery from the patient’s perspective. The duration of the study was from August 2014 to December 2014.

### Study area

Participants for the study were recruited from two tertiary referral hospitals (Manipal Teaching Hospital (MTH) and Western Regional Hospital (WRH)) in Western Nepal. Both of these hospitals had *Aamma Surukshya Karyakram*, literally meaning “Safer Maternity Program” which is operating under the Ministry of Health and Population. The Safer Maternity Program provides both free childbirth services and incentives to women giving birth in a health facility in addition to the incentives to health care worker for each delivery attended [6, 8].

### Sample size and sampling procedure

To estimate the hidden costs of hospital based delivery, 384 post-partum mothers with their husband or house head (284 samples from WRH and 100 samples from MTH) who attended the hospital were interviewed. The interview took place from September 23, 2014 until November 25, 2014. A representative sample size from each health institution was calculated through probability proportionate to size sampling technique. A convenient sampling technique was applied to select the participants (women with their husband or house head). Post-partum mothers were excluded if they did not deliver at the hospital.

### Data collection and analysis

The study questionnaire (S1 File) was developed after reviewing the literatures from Nepal [18], Pakistan [19], Bangladesh [12], Nigeria [20] and Gambia [21]. Of the 30 questions asked to the respondents, half of the questions were adapted from the study conducted in Gambia [21] and remaining half from the studies from Nepal [18] and Bangladesh [12]. The questionnaire was divided into three parts: socio-demographics, delivery related information and delivery related expenditure. The questionnaire was pre-tested with 10% (38/380) of the sample size in Syangja district (a neighboring district) hospital where the same maternity program (*Aama Surakhshya)* was implemented. All questions were asked in Nepali language.

All costs were calculated in Nepalese Rupees (1 US dollar was equivalent to 101.96 Nepalese Rupees during the time of the study). From the patient’s perspective, replacement cost method was used to estimate the hidden costs of hospital based delivery. The cost was calculated based on the market rate for that particular date with the unit cost of a postpartum mother with their husband or house head as a proxy value (or ‘shadow price’) [22]. Caretakers of postpartum mothers were asked to provide medical records and receipts of payments.

Respondents were also asked about their loss of wages during the period of hospital delivery in order to calculate the opportunity cost for days of work loss generated by the condition, assuming that the respondent’s level of earnings reflected productivity (based on the human capital approach) [22]. In this study, loss of wages of the respondent for the delivery period was calculated by the following formula [23];

$$\text{Loss of wages}=\frac{\text{Previous Monthly Income}\;(\text{NRs})}{\text{No. of the days in the Month}}\times \text{Length of Stay}$$

Descriptive statistics were presented as frequency, percentage and mean. Chi-square test was used to analyze the association between various variables with hidden costs. P-value less than or equal to 0.05 was taken for statistical significance.

### Study variables

The hidden costs of delivery services included transportation expenses, food and drinking expenses, communication expenses, cost for laundry, cost for fuel, cost for child care, cost for clothes (sleep wear for baby and women), accessories expenses (expenditure of thermos flask, buckets, mug, soap, mat, toothpaste, oil and toilet papers) and loss of wage (opportunity costs of days of work loss) during the hospital stay. A total hidden cost was measured in US dollars to the same year. We dichotomized it into two categories: <25000 NRs and >25000 NRs, which represented 44.8% and 55.2% of participants respectively. Since the data was normally distributed thus absolute mean was selected as cut off.

Socio-demographic characteristics (age, religion, education level, monthly family income and occupation), delivery related characteristics (place of delivery, number of pregnancy, modes of delivery, distance and travel time from respondents’ house to hospital and length of stay) were analyzed to determine the association with total hidden costs.

### Ethics Statement

This study obtained an ethical approval from the Department of Public Health of La Grandee International College. The study was a part of the thesis for the completion of “Bachelor Public Health” Program in La Grandee International College under Pokhara University. The college has an independent research supervisory department which examines the various issues including ethical issues of research. All research studies are endorsed by college only after the approval from academic committee and research supervisory department. Further approval from both hospitals (Manipal Teaching Hospital and Western Regional Hospital) was obtained before the start of the study. Written informed consent was obtained from all participants who were 18 years or above. For respondents (post-partum mothers) who were below 18 years, written informed consent was obtained from their husbands or household heads with oral consent from respondents. The consent procedure was approved both by the college and the hospitals where study was conducted. The lowest age of post-partum mother in this study was 15 years. No incentives were provided to participants.

## Results

### Demographic characteristics

The mean age was 23.69±4.47 years (range 15 years to 43 years). Majority of respondents 69% (265) had normal vaginal delivery in the study period followed by 31% (119) who underwent C-section delivery as shown in Table 1. The median monthly family income was 15,000 NRs (147.1 USD) per month. Mean duration of stay in hospital for normal vaginal delivery was 4 days (range 2–13 days) and for C-section was 7 days (range 2–19 days). The median duration of hospital stay for both types of delivery was 4 days (range: 2–19 days). Median total cost of hospital based delivery was 28,670 NRs (281.2 USD). The median patient’s expenditure on hospital based delivery was 13% in proportion of annual family income.

**Table 1:** Distribution of respondents by socio-demographic and delivery related characteristics (n = 384).

Characteristics	Number (%)
**Age in years**	
Mean age = 23.69	
Range = 15–43	
**Religion**	
Hindu	315(82)
Buddhist	69 (18)
**Educational status of mothers (n = 384)**	
Literate	313 (81.5)
Illiterate	71 (18.5)
**Educational status of husband (n = 297)**	
Literate	275 (92.6)
Illiterate	22 (7.4)
**Educational status of house head (n = 87)**	
Literate	55 (63.2)
Illiterate	32 (36.8)
**Occupation of mothers (n = 378)**	
Unemployment	301 (79.6)
Employment	77 (20.4)
**Occupation of husband (n = 295)**	
Unemployment	49 (16.6)
Employment	246 (83.4)
**Occupation of house head (n = 88)**	
Unemployment	30 (34.1)
Employment	58 (65.9)
**Monthly family income**	
≤25000 NRs (245.1US $)	287 (74.7)
>25000 NRs (245.1US $)	97 (25.3)
**Distance between hospital and respondents houses**	
≤15 km	197 (51.3)
>15 km	187 (48.7)
**Time needed to reach hospital from respondents houses**	
≤240 minutes	358 (93.2)
>240 minutes	26 (6.8)
**Number of pregnancy**	
1st Time	258 (67.2)
2nd Time	122 (31.8)
3rd Time	4 (1)
**Modes of delivery**	
Normal Vaginal Delivery	265 (69)
C-section	119 (31)
**Length of stay**	
≤5 days	201 (52.3)
>5 days	183 (47.7)
**Total hospital based delivery expenditures**	
Mean = 31175.6 NRs (305.76 USD) and Median = 28670 NRs (281.2 USD)	
**Range of total hospital based delivery expenditures**	
11575 NRs to 65390 NRs (113.5 USD to 641.3 USD)	

101.96 NRs = 1 USD (Exchange rates fixed for 17 December, 2014 by Nepal Rastra Bank).

### Estimation of hidden costs

Hidden expenditures in normal and C-section delivery were 24,817.1 NRs (243.4 USD) and 32,792.2 NRs (321.6 USD) respectively as shown in Table 2. Average total hidden costs was 27,288.5 NRs (267.6 USD) which was 87.5% of total average hospital based delivery expenditure. The mean patient’s expenditure on the food and drinking, clothes and transport were 53.07%, 9.8% and 7.3% in proportion of total hospital based delivery expenditure respectively. It showed expenditures on food and drinking was the major contributor to hidden costs. For normal delivery and C-section, expenditures for food and drinking were 14,840.86 NRs (145.55 USD) and 20,340.38 NRs (199.49 USD) respectively.

**Table 2:** Hidden cost on hospital based delivery by mode of delivery (n = 384)

Normal vaginal delivery without complication			Caesarean section	
Components of care	Mean in NRs	Min-Max. cost	Mean in NRs	Min-Max. cost
Transport expenses	2192.42	300–10000	2488.95	200–21200
Food and drinking expenses	14840.86	1489–35000	20340.38	5000–34000
Communication expenses	241.7	50–800	305.04	100–1000
Cost for laundry	138.75	0–400	138.66	0–350
Cost for fuel	359.02	0–12300	609.07	0–4000
Cost for child Care	1933.58	500–3500	2447.34	800–1000
Cost for clothes	2739.71	350–8000	3781.47	1100–13000
Accessories expenses *	2371.07	150–18000	2681.31	200–5000
**Total hidden cost**				
NRs	24817.1	9300–52500	32792.2	13000–52500
USD	243.4 USD		321.6 USD	
Mean = 27288.5 NRs (267.6USD)				
Median = 26500 NRs (259.9 USD)				
Range = 9300 NRs to 52500 NRs (91.2 USD to 514.9 USD)				

*Accessories expenses consists cost of given items i.e. thermos flask, buckets, mug, soap, mat, toothpaste, oil and toilet papers.

101.96 NRs = 1 USD (Exchange rate for 17 December, 2014 by Nepal Rastra Bank).

Table 3 shows that the mean loss of wages of mother, husband and house head were 5,963.7 NRs (58.4 USD), 7,429.3 NRs (72.9 USD) and 6,175.9 NRs (60.6 USD) respectively. Similarly, for postpartum women with their husband or house head, the total mean opportunity cost of days of work loss was 8,582.2 NRs (84.1USD) and 8,354.04 NRs (81.9 USD) for normal delivery and C-section respectively.

**Table 3:** Loss of wage (opportunity cost of days of work lost) during delivery period (n = 384)

Variable	Mothers	Husbands	House Heads
Mean lost earning	5963.7 NRs	7429.3 NRs	6175.9 NRs
	(58.4 USD)	(72.9 US $)	(60.6 US $)
**Opportunity cost of days of work lost**	**Mean**	**Median**	**Range**
Normal vaginal delivery without complication	8582.2NRs (84.1USD)	7225.8 NRs (70.8 USD)	89 NRs—59225.8NRs (0.8 USD—580.8 USD)
C-section	8354.04NRs (81.9 USD)	6967.7NRs (68.33 USD)	186 NRs—25161.2NRs (1.8USD—246.7 USD)

101.96 NRs = 1 USD (Exchange rate for 17 December, 2014 by Nepal Rastra Bank).

### Association between total hidden costs and other characteristics

Table 4 shows that there was a statistically significant association among places of delivery (p = 0.0001), modes of delivery (p = 0.0001), length of stay in hospital (p = 0.0001) and distance between respondent’s house and hospital (p = 0.0001) with the hidden costs on hospital based delivery. Similarly, there was a significant association between educational status of mothers (p = 0.007), occupation of house head (p = 0.011) family monthly income (p = 0.014) and travel time between respondent’s house and hospital (p = 0.007) with the hidden costs of delivery services provided by the hospital.

**Table 4:** Association of hidden cost on delivery services with study variables (n = 384).

Characteristics	Hidden cost on delivery services		P-value
	≤ 25,000 NRs n = 172 (44.8%)	>25,000 NRs n = 212 (55.2%)	
**Age of respondents**			
≤ 25 Years	101 (43.9)	129 (56.1)	0.677
> 25 Years	71 (46.1)	83 (53.9)	
**Religion of respondents**			
Hindu	138 (43.8)	177(56.2)	0.408
Buddhist	34 (49.3)	35 (50.7)	
**Educational status of mother**			
Illiterate	42 (59.2)	29 (40.8)	0.007*
Literate	130 (41.5)	183 (58.5)	
**Educational status of husband**			
Illiterate	10 (45.5)	12 (54.5)	0.843
Literate	119 (43.3)	156 (56.7)	
**Educational status of house head**			
Illiterate	8 (25)	24 (75)	0.058
Literate	25 (45.5)	30 (54.5)	
**Occupation of mother**			
Unemployment	136 (45.2)	165 (54.8)	0.714
Employment	33 (42.9)	44 (57.1)	
**Occupation of husband**			
Unemployment	25 (51)	24 (49)	0.198
Employment	101 (41.1)	145 (58.9)	
**Occupation of house head**			
Unemployment	21 (70)	9 (30)	0.011*
Employment	24 (41.4)	34 (58.6)	
**Family monthly income**			
>25000 NRs	33(34)	64(66)	0.014*
≤ 25000 NRs	139(48.4)	148 (51.6)	
**Places of delivery**			
Private hospital	2 (2)	98 (98)	0.0001*
Public hospital	170 (59.9)	114 (40.1)	
**Numbers of pregnancy**			
One	110 (42.6)	148 (57.4)	0.477
Two	60 (49.2)	62 (50.8)	
Three	2 (50)	2 (50)	
**Modes of delivery**			
NVD	145 (54.7)	120 (45.3)	0.0001*
C-section	27 (22.7)	92 (77.3)	
**Length of stay in hospital**			
≤ 5 days	150 (74.6)	51 (25.4)	0.0001*
> 5 days	22 (12)	161 (88)	
**Distance between respondents houses and hospital**			
≤15 km	107 (54.3)	90 (45.7)	0.0001*
> 15 km	65 (34.8)	122 (65.2)	
**Travel time between respondents’ house and hospital**			
≤240 minutes	167 (46.6)	191 (53.4)	0.007*
> 240 minutes	5 (19.2)	21 (80.8)	

*Significant by Chi-square.

## Discussion

To our knowledge, this is the first study in Nepal assessing the hidden costs during hospital delivery. In this study, the average total hidden costs was 27,288.5 NRs (267.6 USD) which was 87.5% of total average hospital based delivery expenditures (31,175.6 NRs). These findings suggest that hospital based delivery can be an expensive experience for women from lower socio-economic status as this study shows that the median patient’s expenditure on hospital based delivery (28,670 NRs) was 13% in proportion of median annual family income (15,000 NRs). Postpartum mothers who came with their husband or house head in this study were economically vulnerable than in the study conducted in the public hospital of Bangladesh [24] where median patient expenditure was equivalent to 7% of annual household income. The discrepancy could have been because of the study conducted in Bangladesh was older than ours. An earlier study conducted in 8 districts of Nepal [1] found a total hidden costs of 4,622 NRs (61.1 USD) for a normal delivery that was 87.2% of total hospital costs which has been inconsistent with our study because in our study, expenses on various new items were included in the hidden costs of maternity services which can increase the expenditure of hospital delivery.

The patient’s mean expenditure on food and drinking (53.07%) was higher than for clothes (9.8%) and transportation (7.3%). The finding in this study is unique as (other) studies from different parts of the world showed different lists of expenses. In a study conducted at government facilities in Bangladesh [12], costs for medicine, transportation and food were the first, second and third major expenditure of hospital based delivery respectively. Similarly, expenditures on child care and food for patients were first and second major expenditures of hospital based delivery in a study done in Nigeria [20]. Unlike the studies in Bangladesh and Nigeria, our study showed the expenditure on food and drinking as a major contributor to hidden costs. This finding implies the need of focus for service providers to hospital attenders.

The mean opportunity cost of days of work loss (loss of wages) for normal delivery and C-section were 8,582.2 NRs (84.1 USD) and 8,354.04 NRs (81.9 USD) respectively which showed that loss of wages for pregnant women with their husband or house head during delivery was one of the major contributors to the hidden costs. A previous study in Nepal [1] found a lower opportunity cost for normal delivery and C-section delivery which was 492 NRs (6.5 USD) and 1,660 NRs (21.9 USD) respectively. The difference in opportunity cost between previous study and this study could have been because this study reflects the urban hospital’s context where costs of travelling, food and living are higher than in the district level hospitals. Similarly, our study accounted the opportunity cost for both pregnant women and the caretakers.

To reduce negative externalities from hospital based delivery, this study showed that hidden costs need to be reduced by focusing on several socio-demographic factors (education among the pregnant women, occupational status of house hold head and family economic status) and delivery related factors (place and modes of delivery, length of stay, distance from hospital and travel time). The findings in this study are consistent with studies conducted in Lao People's Democratic Republic [25], Greece [26], Pakistan [19] and Nepal [18] which showed occupational status, educational level, monthly family-income, mode of delivery, place of delivery and length of stay at hospital, were associated with hidden charges.

Furthermore, this study found that none of the factors like age of respondents, religion, educational status of husband, educational status of house head, occupation of husband, occupation of house head and number of pregnancies affected the hidden costs on the delivery services (p>0.05).

This study may not represent the more rural communities as the study was conducted in tertiary health facilities in an urban area, however, tertiary health care centers selected in this study are attended by participants from various regions including rural regions. The generalizability of this study is therefore, partially restricted to urban settings. Further studies are recommended to conduct in rural settings such as district hospital in hilly and mountainous regions that might better reveal the rural hospital attendance. Besides hidden costs estimation, intangible costs which reflect the pregnant women and the relatives’ psychosocial stresses to travel to the hospital and stay at hospital were beyond the scope of this study.

## Conclusion

Experiences of hidden costs on hospital based delivery and opportunity cost of days of work loss were high and postpartum mothers with their husband or house head were economically affected. Half of the hidden costs were attributed to food and drinking. Hidden costs and opportunity cost could be a significant barrier to hospital delivery for women from poor household and women from remote regions. Education of mothers, occupation of house head, family monthly income, mode of delivery, place of delivery, length of stay in the hospital, distance from house to hospital, travel time from house to hospital were factors associated with the hidden cost on hospital based delivery. Current delivery services need to take in account hidden costs/expenditures for pregnant women and their relatives. Current remuneration (10–70 USD) needs substantial revision to account the hidden costs of hospital based delivery by increasing it to 250 USD for normal delivery and 350 USD for C-section. Decentralization of the obstetric care to remote and under-privileged population might reduce the economic burden of pregnant women attending health care facilities.

## Supporting Information

S1 File Questionnaire. (PDF)
