# Supplementary material for: Hidden Costs of Hospital Based Delivery from Two Tertiary Hospitals in Western Nepal
Source: PLoS One. 2016 Jun 16;11(6):e0157746. doi: 10.1371/journal.pone.0157746 (PMC4911061; doi:10.1371/journal.pone.0157746)
Supplement: S1 File — (PDF) [file pone.0157746.s001.pdf]

Questionnaire

“Hidden Cost of Hospital Based Delivery in two Tertiary Hospitals of Western, Nepal”

A. In English language

Section A

Socio-demographic Information

**Q. No**	**Socio Demographic Characteristics**	**Option**			**Coding**	**Remark**
1.	Age	…………………				
2.	Religion	a. Hindu<br>b. Buddhist<br>c. Muslim<br>d. Christian<br>e. Others (specify)……………………… ……………………			1<br>2<br>3<br>4<br>5	
3.	Education		**Mother**	**Husband /HH**	**Tick who are present**	
		Illiterate<br>Literate<br>Primary class<br>Lower secondary<br>Secondary<br>Higher secondary			1<br>2<br>3<br>4<br>5<br>6	
4.	What is the monthly income of your family?	……………NRs.				
5.	What is your regular work/employment?		**Mother**	**Husband /HH**		If housewife or unemployed – go to question 6
		Unemployed<br>Government<br>Job			1<br>2<br>3	

		Private Job<br>Farmer/farmers wife<br>Self-employed<br>Housewife<br>Other (please specify)……<br>……………			 4<br>5<br>6<br>7	

Section B

Delivery Related Information

Q. No	Delivery Related Characteristics	Option	Coding	Go
6.	Which number of pregnancy is it?	………………..		
7.	What type of delivery was it?	Normal, vaginal delivery without complication<br>Forceps or vacuum extraction or episiotomy<br>C- section<br>Complication<br>Other (specify)…………………………	1<br><br>2<br>3<br>4<br><br>5	
8.	What is the distance between your house and hospital?	………………….km.		
9.	How long did it take you to reach hospital?	…….Hours and ………Minutes […….By Transportation ………….By Foot]		
10.	When are you going to be discharged from the hospital?	……………days after delivery		

Section C

Maternity Care Expenditure

A. Visible Cost

S.N	Do you pay for the given items?	Visible Cost	Price in NRs
1.	**….**No (0)<br>…Yes (1)	Medicine purchase from private pharmaceutical	………NRs
2.	**….**No (0)<br>…Yes (1)	Other medical material (Sutures, IV sets etc)	………NRs
3.	**….**No (0)<br>…Yes (1)	Operations	………NRs
4.	**….**No (0)<br>…Yes (1)	Bed price	………NRs
5.	**….**No (0)<br>…Yes (1)	Dressings and bandages	………NRs
6.	**….**No (0)<br>…Yes (1)	Needles and syringes	………NRs
7.	**….**No (0)<br>…Yes (1)	Blood transfusion	………NRs
8.	Others (please specify)…………..		………NRs
	Total		/______/ NRs

B.
Hidden Cost

S. N.	Items	No. of Person	Preliminary Cost	Frequency	Total amount	Remark
1.	Transportation					
2.	Food and drink expenses					
3.	Communication					
4.	Gift to the Staffs					
5.	Laundry					
6.	Tips					
7.	Other (specify)………… …………………					

Thank you very much for your time!

B. In Nepali Language

खण्ड (क)

(सामाजिक तथा जनसांङखिक विवरण)

<table>
<tr><td>प्र.नं</td><td>सामाजिक तथा जनसांङखिक गुण</td><td colspan="3">तह</td><td>कोडिङ्ग</td><td>जाने</td></tr>
<tr><td>१.</td><td>उमेर (पुरा गरेको वर्ष)</td><td colspan="3">……………………………</td><td></td><td></td></tr>
<tr><td>२.</td><td>धर्म</td><td colspan="3">क. हिन्दु
ख. बौद्ध
ग. इस्लाम
घ. क्रिश्चियन
ङ. अन्य
(खुलाउनुहोस्)……………………………</td><td>१
२
३
४
५</td><td></td></tr>
<tr><td rowspan="2">३.</td><td rowspan="2">शैक्षिक स्थिति</td><td></td><td>श्रीमति</td><td>श्रीमान् वा घरमूलि</td><td>वर्तमानमा भएकालाई चिन्न लगाउनु</td><td rowspan="2"></td></tr>
<tr><td>निरक्षर
सारक्षर
प्राथमिक विद्यालय
निम्न माध्यमिक विद्यालय
माध्यमिक विद्यालय
उच्च माध्यमिक</td><td></td><td></td><td>१
२
३
४
५
६</td></tr>
<tr><td>४.</td><td>तपाईको परिवारको मार्सिक आम्दानी कति हो ?</td><td colspan="3">…………………ने.रु.</td><td></td><td></td></tr>
<tr><td>५.</td><td>तपाईको घरको मुख्य आम्दानीको स्रोत के हो ?(बहु उत्तर आउन सक्छ)</td><td colspan="3">सरकारी जागिर
गैह्र सरकारी संस्था
ब्यापार
श्रम
कृषि
अन्य (खुलाउनुहोस्)……………………</td><td>१
२
३
४
५
६</td><td></td></tr>
</table>

खण्ड (ख)

(सुत्केरी सम्बन्धि विवरण)

प्र.नं	सुत्केरी सम्बन्धि गुण	तह	कोडिङ्ग	जाने
६.	कतियौ गर्भ थियो यो ?	………………………………		
७.	कस्तो प्रकारको प्रसुति थियो ?	समान्य, खर्तरा मुत्त<br>भ्याकुमले तान्ने<br>अपरेसन<br>खर्तरा<br>अन्य (खुलाउनुहोस्)………………………	१<br>२<br>३<br>४<br>५	
८.	तपाईको घरबाट हस्पिटलसम्मको दुरी कति छ ?	……………कि. मि.		
९.	तपाईलाई हस्पिटल पुग्न कति समय लाग्यो ?	……….घण्टा……….मिनेट<br>ृसवारी साधनबाट………पैदलबाट……`	जूनबाट तिनीहरु पुगे त्यसमा चिन्न लगाउनु	
१०.	हस्पिटलबाट कति दिनमा डिस्चार्ज हुनु हुन्छ ?	………….दिन सुत्केरी भएपछि		

खण्ड (ग)

सुत्केरी स्याहार खर्च

(क) देख्न सक्ने खर्च

क्र.सं.	के खर्च गर्नुभयो ?	देख्न सक्ने खर्च	ने.रु.
(१)	गरिएन (०) …………<br>गरियो (१) …………	औषधि बाहिरको मेडिकल पसलबाट किनेको	…………………………
(२)	गरिएन (०) …………<br>गरियो (१) …………	अन्य मेडिकल सामान (सुचर, आइ.वि.सेट आदि)	…………………………
(३)	गरिएन (०) …………<br>गरियो (१) …………	अप्रेशन	…………………………
(४)	गरिएन (०) …………<br>गरियो (१) …………	बेड चार्ज	…………………………
(५)	गरिएन (०) …………<br>गरियो (१) …………	ड्रेसिङ्ग	…………………………
(६)	गरिएन (०) …………<br>गरियो (१) …………	सुई	…………………………
(७)	गरिएन (०) …………<br>गरियो (१) …………	रगत लिने	…………………………
(८)	अन्य (खुलाउनुहोस्)………………………		…………………………

जम्मा	ने.रु.........................

(ख) अदृश्य खर्च

क्र.सं.	किसिम	कति जनालाई	प्रारम्भिक खर्च	कति चोटी	एकमुष्ठ रकम	कैफियत
(१)	सवारी साधन					
(२)	खाना खर्च					
(३)	सूचना संचार खर्च					
(४)	कर्मचारीलाई चिनो					
(५)	कपडाको सरसफाई					
(६)	बकस/टिप्स					
(७)	अन्य (खुलाउनुहोस)					
